# The Two-Component Regulatory System VicRK is Important to Virulence of *Streptococcus equi* Subspecies *equi*

**DOI:** 10.2174/1874285800802010089

**Published:** 2008-06-19

**Authors:** Mengyao Liu, Michael J McClure, Hui Zhu, Gang Xie, Benfang Lei

**Affiliations:** Department of Veterinary Molecular Biology, Montana State University, Bozeman, Montana 59717, USA

## Abstract

This study aims at evaluating the importance of the two-component regulatory system VicRK to virulence of the horse pathogen *Streptococcus equi* subspecies *equi* and the potential of a *vicK* mutant as a live vaccine candidate using mouse infection models. The *vicK* gene was deleted by gene replacement. The Δ*vicK* mutant is attenuated in virulence in both subcutaneous and intranasal infections in mice. Δ*vicK* grows less slowly than the parent strain but retains the ability of *S. equi* to resist to phagocytosis by polymorphoneuclear leukocytes, suggesting that the *vicK* deletion causes growth defect. Δ*vicK* infection protects mice against reinfection with a wild-type *S. equi* strain. Intranasal Δ*vicK* infection induces production of anti-SeM mucosal IgA and systemic IgG. These results indicate that VicRK is important to *S. equi* growth and virulence and suggest that Δ*vicK* has the potential to be developed as a live *S. equi* vaccine.

## INTRODUCTION

Bacterial pathogens produce many two-component regulatory systems to regulate gene expression by specific environmental signals [1]. These systems consist of membrane protein sensors and cognate cytoplasmic response regulators. The regulator is phosphorylated by the sensor in response to a specific signal, activating or repressing the transcription of target genes. The two-component regulatory system VicRK or YycFG is specific for Gram-positive bacteria. The regulator component VicR is essential in *Bacillus subtilis* [2], *Staphylococcus aureus* [3], and *Streptococcus pneumoniae* [4-5] but appears not to be essential in *Streptococcus pyogenes* [6]. The deletion of the *vicK *gene can be readily inactivated in *S. pneumoniae* [7], *Streptococcus mutans* [8], and *S. pyogenes* [6] but not in *B. subtilis *[2] and *S. aureus* [3]. Conditional and unconditional *vicRK* mutants display various phenotypes, including defects in morphology and cell wall synthesis, decreased competence, sensitivity to antibiotics and fatty acids, defects in biofilm formation, and attenuated virulence, growth defect, and sensitivity to osmotic pressure [3, 6, 8-11].

The *vicRK* system of Gram-positive bacterium *Streptococcus equi *subspecies* equi* (*S. equi*) has not been studied. This pathogen causes equine strangles, a highly contagious purulent lymphadenitis [12-13]. The infection initially causes nasal discharge and fever and, then, leads to abscess formation in local lymph nodes, causing respiratory difficulty. Although the infection at the lymph nodes cause massive infiltration of polymorphoneuclear leukocytes (PMNs) [14], *S. equi* resists phagocytosis by PMNs and rapidly multiplies, forming an abscess of large numbers of degenerating PMNs and long chains of *S. equi* [15]. The hyaluronic acid capsule and *S. equi* M-like protein (SeM) are both required for the resistance to phagocytosis by PMNs [16-17]. Most horses recovered from strangles have immunity against *S. equi* reinfection for up to 5 years [18]. It is believed that the immunity is mediated by mucosal antibodies specific to SeM and other protective antigens. An intranasal vaccine made of live attenuated strain has been used in USA, which lacks the hyaluronic acid capsule, and various adverse effects, including pharyngeal lymphadenopathy, limb edema, and bastard strangles abscesses, have been reported [15].

This study aims at evaluating the importance of VicRK to *S. equi* virulence and the potential of a *vicK* deletion mutant as a live vaccine using mouse infection models. We found that the *vicK* deletion attenuated *S. equi* virulence in mouse models of subcutaneous and intranasal infections and that infection with a *vicK* deletion mutant confers protection against subsequent infection with wild-type *S. equi* and induces production of mucosal and systemic immunoglobins to SeM in nasal infection.

## MATERIALS AND METHODS

### Bacterial Strains and Growth

*S. equi* strain SEM1 was isolated in 2003 from a horse with strangles in Montana, USA. SEM1 and its mutant were routinely grown in Todd-Hewitt broth supplemented with 0.2% yeast extract (THY) in 5% CO_2_ at 37°C without and with 150 mg/liter spectinomycin, respectively.

### Generation of a *vicK* Deletion Mutant

**Fig. (1):**
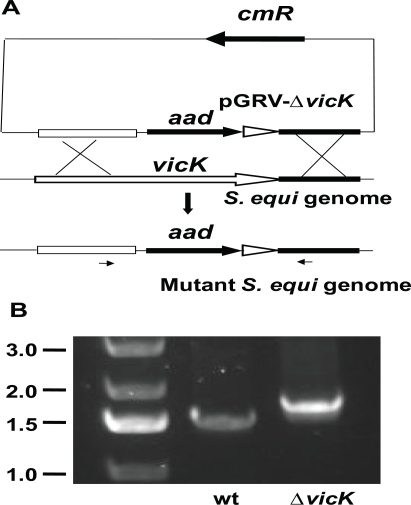
Deletion of the vicK gene. **A)** schematic for *vicK* deletion by gene replacement. The two flanking fragments of the internal *vicK* fragment to be deleted were cloned into the up- and down-stream ends of the aad gene in pGRV. The resulting plasmid pGRV-Δ*vicK* was introduced into *S. equi*, and double crossover in the homologous regions between the plasmid and *S. equi* genome resulted in ΔvicK mutants. **B)** PCR confirmation of the vicK dele-tion. The picture shows agarose gel analysis of PCR reactions using mutant (lane Δ*vicK*) or wild-type (lane wt) genomic DNA as tem-plate and primers indicated by the arrows under the mutant genome

A *vicK* deletion mutant (Δ*vicK*) of *S. equi* SEM1 was generated by gene replacement (Fig. **1**). The upstream and downstream flanking fragments of the deleted internal fragment (bases 451-1282) of *vicK* were PCR-amplified using paired primers 5’- GAAGCTTCTTATGACTAAGGACATCATTGGAAC-3’/5’-GAGATCTGGTGTAAGGTGAGTC ACTGTC-3’ and 5’-AGGATCCCCTTTACCATTGTG TTA CCTTACG-3’/5’-AGTCGACCCTGTATCCGTCAGCATG AC-3’, respectively. The PCR products of the upstream and downstream fragments were sequentially cloned into pGRV [19] at the *Hind*III/*Bgl*II and *Bam*HI/*Sal*I sites, respectively, to yield pGRV-Δ*vicK*. pGRV-Δ*vicK* was introduced into *S. equi* strain SEM1 by electroporation using the conditions described previously for *S. pyogenes* [6], and the sample was plated on THY agar plate with 150 mg/liter spectinomycin. The obtained colonies, which could be derived from a single or double crossover recombination, were screened by PCR using primers 5’-GAGACTGCTC AAAAGCAGCTC-3’ and 5’-GATTTGACTCAATCAAGTAGC-3’ and DNA sequencing analyses to identify the desired deletion mutant.

### S. equi Growth in Rabbit Blood

*S. equi *strains were harvested at the exponential growth phase, washed three times with pyrogen-free Dulbecco’s phosphate-buffered saline (DPBS), and inoculated at 2 x 10^4^ cfu/ml in heparinized rabbit blood. The samples were rotated end-to-end at 37°C for 4 h, and numbers of viable *S. equi* in the samples and actual inocula were determined by plating on THY agar. Growth factor is defined as the ratio of colony-forming units (cfu) of each sample after 4-h incubation to cfu in the inoculum.

### Phagocytosis Assay

Phagocytosis assay was performed as described previously [6, 20]. Briefly, *S. equi* SEM1 wild-type and Δ*vicK* cells from exponential growth phase in THY were washed with phosphate-buffered saline (PBS) and labeled with 0.75 µg/mL FITC in PBS at 37°C for 20 min. The labeled bacteria were washed and suspended at 1 x 10^9 ^cfu/ml in PBS. Ten μl of the labeled bacteria were mixed with 100 μl of non-immune heparinized rabbit or horse blood and incubated with gentle shaking at 37(C for 5 or 15 min. The samples were immediately processed using an Immunolyse Kit (Beckman Coulter) according to the manufacturer’s protocol and analyzed by flow cytometry. The percentage of PMNs with fluorescent bacteria was used as a measure of phagocytosis efficiency.

### Mouse Infections

*S. equi* strains were harvested at exponential phase, washed with DPBS, and inoculated subcutaneously or intranasally at inocula specified in figure legends into groups of 8 female outbred CD-1 Swiss mice. Survival rates were examined daily for 20 days after inoculation. At the end of the intranasal infection experiment, blood was collected from the surviving mice via cardiac puncture, and the nasal wash fluids were then obtained as follows. The trachea was perforated, a small tube was inserted into the opening, and the nasal cavity was slowly flushed with 1.0 ml DPBS through the tube. All animal procedures were approved by the Institutional Animal Care and Use Committee at Montana State University, Bozeman, USA.

### Enzyme-linked Immunosorbant Assay (ELISA) and Western Blotting Analysis

Relative levels of anti-SeM IgG in sera of mice recovered from the intranasal *S. equi* infections were estimated by ELISA and Western blotting using a truncated recombinant SeM containing amino acids 38 to 260 (SeM^38-260^) with described procedures [21]. Briefly for ELISA, microtiter plates were coated overnight with SeM^38-260^ at a concentration of 0.25 µg/well. The plates were washed four times with PBS containing 0.1% (vol/vol) Tween 20 (PBS-T), blocked with 0.1% bovine albumin (BSA) in PBS-T for 2 h at room temperature, and washed as described above. The plate was incubated with 100 μl/well of mouse sera diluted at 1:100 to 1:51200 in 0.1% BSA in PBS-T and washed as described above. The wells were incubated with goat anti-mouse IgG (H + L)-peroxidase conjugate (1:4,000 dilution). The plates were washed as described above and washed four times with PBS to remove Tween 20. The plates were developed with 100 µl/well of ABTS solution for 30 min and the absorbance was measured at 405 nm. Titers were determined by the geometric method. The presence of SeM^38-260^-specific IgA in the nasal wash samples were determined by A_405_ in the ELISA assay as described above using 100 μl of 2-fold-diluted nasal wash samples and goat anti-mouse IgA HRP conjugate (Bethyl Laboratories, Inc.).

## RESULTS

### S. equi vicK Deletion Mutant

The *S. equi* *vicRK* genes were found by blasting the *S. equi* genome database (http://www.sanger.ac.uk/Projects/S_equi ) with the *S. pyogenes vicRK* sequence. Gene replacement strategy was used to generate *vicK*-deletion mutant (Fig. **1A**). The vector pGRV has two genes *aad* and *cmR* for selections with spectinomycin and chloramphenicol, respectively. The two upstream and downstream flanking fragments of the internal *vicK* fragment from Tyr151 to Ser427 to be deleted were cloned at the upstream and downstream ends of the *aad* gene, respectively, resulting in suicide plasmid pGRV-Δ*vicK*. Single crossover between one flanking fragment in pGRV-Δ*vicK* and the homologous region in the genome would lead to the insertion of the whole plasmid into *S. equi* genome, resulting in strains resistant to both spectinomycin and chloramphenicol. Double crossover**at both of the flanking fragments would lead to the replacement of the *vicK* internal fragment with the *aad* gene, resulting in *vicK* deletion strains with resistance only to spectinomycin. The colonies on spectinomycin agar plates were tested for resistance to chloramphenicol. Three putative Δ*vicK* strains, which were spectinomycin-resistant and chloramphenicol-sensitive, were obtained. PCR analyses using the primers located beyond the deleted fragment resulted in the PCR product from these strains that were expectedly bigger than that from the wild-type strain because the replacing fragment was bigger than the displaced *vicK* fragment (Fig. **1B**). DNA sequencing confirmed the desired deletion. One deletion strain was randomly chosen for further characterization.

### Growth of *ΔvicK* in THY and Rabbit Blood

**Fig. (2):**
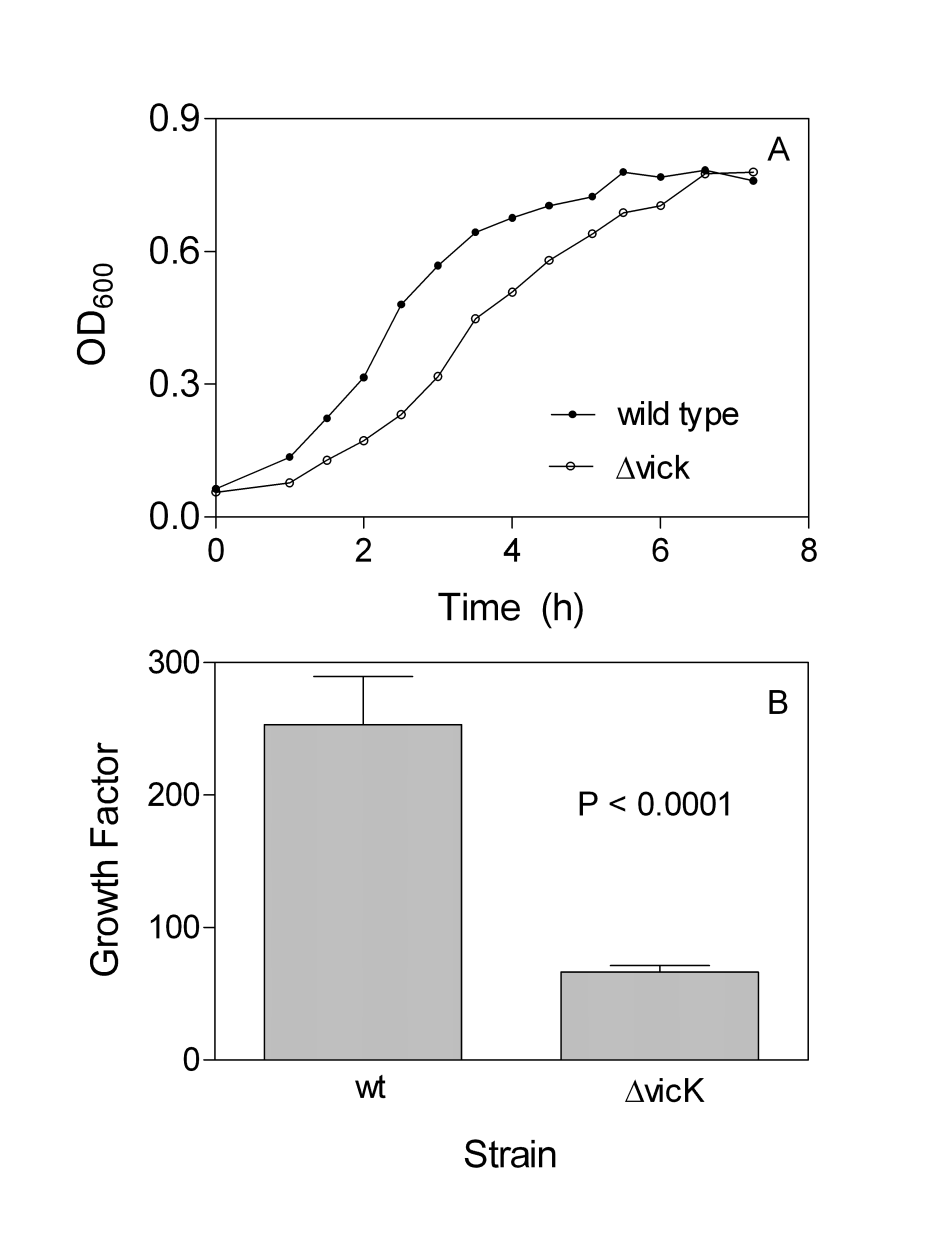
**A)** growth curves of wild-type and Δ*vicK* strains in THY.Cultures at the exponential phase were inoculated into fresh THY,and OD_600_ was measured at the indicated times. **B)** growth of wildtype and ΔvicK strains in rabbit blood. Approximately 2 x 10^4^ cfu of each strain was inoculated into 1 ml blood in triplicate. Numbers of the bacteria in inocula and in the samples after end-to-end rotation at 37°C for 4 h were determined by plating. The growth factor (cfu at 4 h/ cfu at 0 h) ± SD is presented.

The growth curve of the Δ*vicK* mutant in THY displays a longer early growth phase and smaller slope in the exponential growth phase than that of the parent strain (Fig. **2A**), indicating that the *vicK* deletion detrimentally affects *S. equi* growth. The effect of the deletion on *S. equi* growth in blood was also examined. The wild-type and Δ*vicK* *S. equi* strains were inoculated into 1 ml heparinized rabbit blood at an inoculum of approximately 20,000 cfu. The samples were incubated for 4 h, and the numbers of the bacteria in the samples and inocula at time zero were determined by plating. The growth factors, the ratio of cfu in the sample at 4 h over cfu at time zero, were 250 and 66 for the wild type and Δ*vicK* strains, respectively (Fig. **2B**). Thus, the Δ*vicK* mutant has significantly reduced ability to grow in rabbit blood (P <0.0001).

### No Effect of the *vicK* Deletion on Resistance of *S. equi* to Phagocytosis by PMNs

**Fig. (3):**
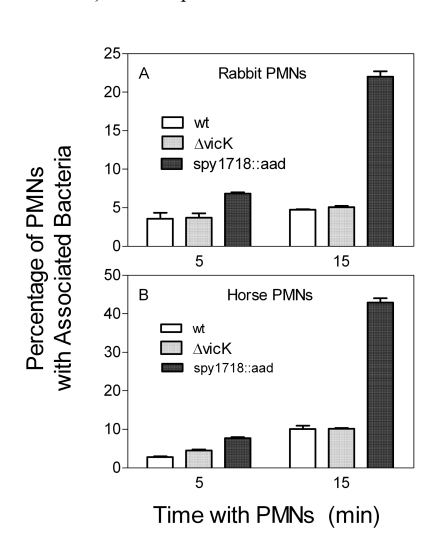
Association of wild-type and Δ*vicK* bacteria with rabbit **(A)**and horse **(B)** PMNs. FITC-labeled bacteria (10^7^ cfu) were incubated with 100 µl heparinized blood at 37°C for 5 or 15 min. Red blood cells were lysed using an Immunolyse kit, and percentages of PMNs with associated (bound and phagocytosed) bacteria determined by flow cytometry are presented. A *S. pyogenes spy1718::aad* mutant was included as a positive control.

To determine whether the ΔvicK deletion affects the resistance of S. equi to phagocytosis by PMNs, the phagocytosis of wild-type and ΔvicK bacteria by PMNs in non-immune horse and rabbit blood was compared. FITC-labeled wild-type S. equi, ΔvicK mutant, and S. pyogenes spy1718::aad mutant were incubated with heparinized horse or rabbit blood for 5 and 15 min, and percentages of PMNs associated with fluorescent bacteria were quantified using flow cytometry analysis. The spy1718::aad mutant of S. pyogenes, which is no longer resistant to phagocytosis by PMNs, was used as a positive control in the assay. The percentages of PMNs with associated wild-type S. equi and spy1718::aad were low and high, respectively, indicating that the assay worked well to evaluate resistance of the bacteria to phagocytosis. There was no significant difference in the percentages of PMNs associated with wild-type and ΔvicK bacteria at both time points and in both horse and rabbit blood (Fig. **3**), indicating that the ΔvicK mutant retains the ability of S. equi to resist to phagocytosis by PMNs.

### Attenuation of *S. equi* Virulence by *vicK* Deletion

**Fig. (4):**
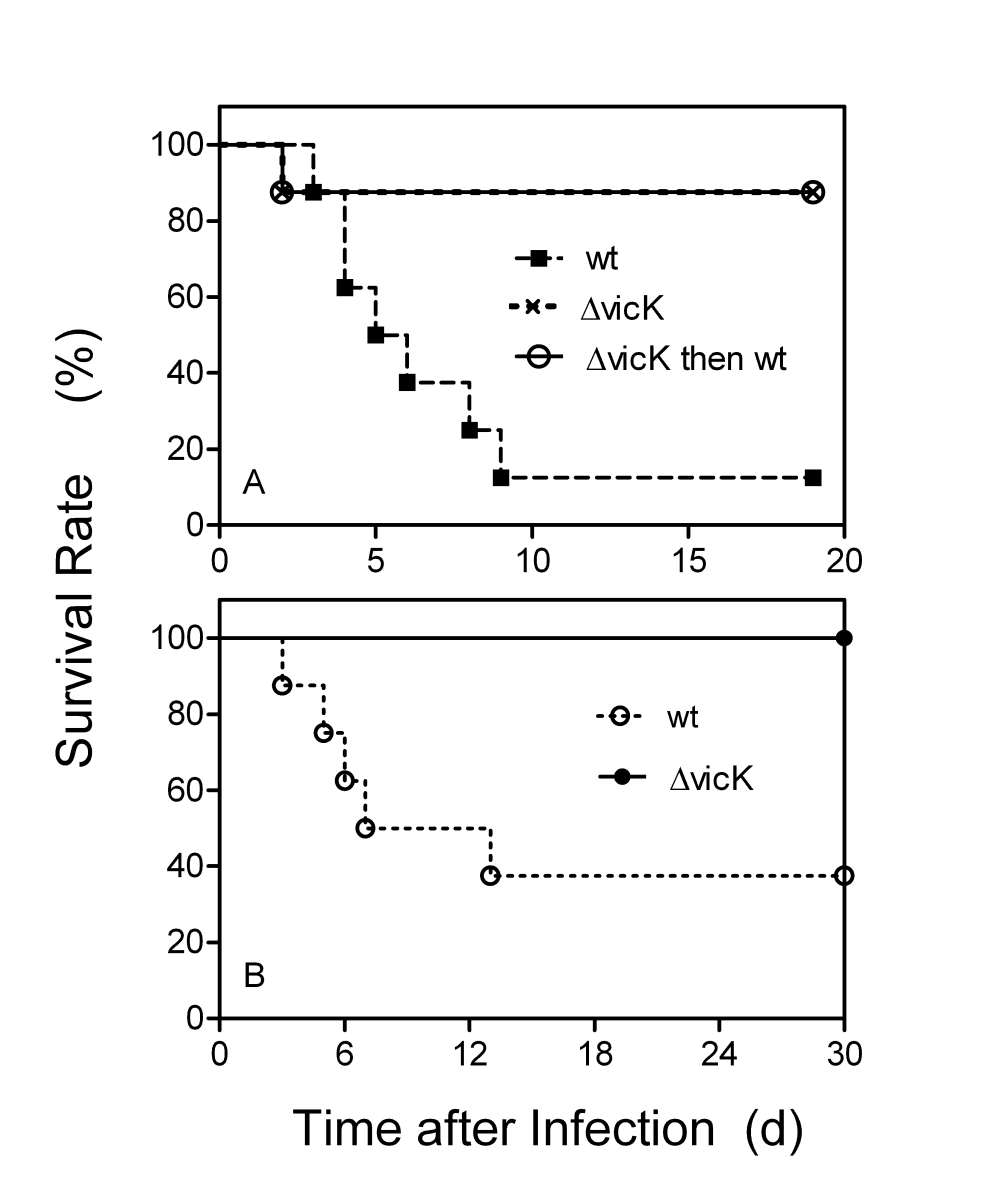
Attenuation of *S. equi* virulence by *vicK* deletion and protection of mice against wild-type *S. equi* infection conferred by preceding Δ*vicK* infection. Groups of 8 CD-1 mice were inoculated subcutaneously **(A)** and intranasally **(B)** with 1.1 x 108 cfu of each strain, and survival rates were determined daily. Panel A also includes the results of the subcutaneous infection with 1.5 x 108 cfu of the wild-type strain on the 7 mice survived from the subcutaneous vicK infection (the open circles)

Group of 8 mice were subcutaneously inoculated with 1.1 x 10^8^ cfu wild-type or Δ*vicK* mutant strains. Seven of the 8 mice infected with the wild-type *S. equi* strain died, whereas 7 of the 8 mice inoculated with Δ*vicK* survived (Fig. **4A**). The infection was performed in a model of intranasal infection as well. All the 8 mice infected with Δ*vicK* survived, whereas 5 of the 8 mice infected with the wild-type *S. equi* strain died (Fig. **4B**). These results indicate that the *vicK* deletion significantly attenuated *S. equi* virulence in both mouse models of subcutaneous (P = 0.0066) and nasal (P = 0.0085) infections.

### Δ*vicK* Infection Confers Protection of Mice against Reinfection with Wild-Type *S. equi*

To test whether Δ*vicK* infection confers immunity against *S. equi* infection, the seven mice recovered from the subcutaneous Δ*vicK* infection was reinfected subcutaneously with 1.5 x 10^8^ cfu wild-type *S. equi* 30 days after the first infection and monitored for 18 days. Six of the 7 mice survived the reinfection (Fig. **4A**), suggesting that the Δ*vicK* infection induces immunity against *S. equi* infection.

### Intranasal Δ*vicK* Infection Induces SeM-Specific Mucosal IgA and Systemic IgG

**Fig. (5):**
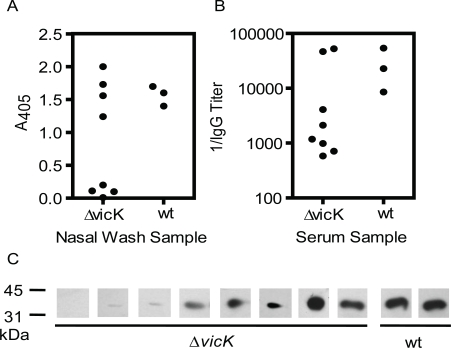
Assessment of SeM-specific mucosal IgA and systemic IgG production in mice with the intranasal *S. equi* infection by ELISA and Western immunoblot. Nasal wash and serum samples were collected from the surviving mice in the nasal infections with wild-type and ΔvicK strains of Fig. 4B on day 30 after inoculation. **A)** immunoreactivity of SeM38-260 with IgA in the nasal wash samples. **B)** the reciprocal of anti-SeM^38-260^ IgG titers in the serum samples. **C)** immunoblots demonstrating anti-SeM^38-260^ IgG in the serum samples, which were diluted by 1:1000 in the assay.

To examine the humoral immune responses in the intranasal Δ*vicK* infection, nasal wash and serum samples were collected from the 8 mice infected intranasally with Δ*vicK* and 3 surviving mice infected with the wild-type *S. equi* 30 days after infection. Half of the nasal wash samples from the mice infected with Δ*vicK* had similar levels of SeM^38-260^-IgA reactivity with those from the mice infected with the wild-type strains. Similarly, these 4 mice with higher IgA levels also had higher levels of SeM-specific systemic IgG (Fig. **5B**). Western blotting analysis was used to confirm the presence of SeM-specific IgG. The wild-type sera and 5 of the 8 Δ*vicK* samples had strong immunoreactions with SeM^38-260^ in Western blotting analysis (Fig. **5C**). Thus, the Δ*vicK* mutant has the ability to induce mucosal and systemic immune responses, though there was host variation in these responses caused by Δ*vicK* infection.

## DISCUSSION

VicK is essential in *B. subtilis* [2] and *S. aureus* [3] but not in *S. pneumoniae *[7], *S. mutans *[8], and *S. pyogenes *[6]. We successfully deleted the *vicK* gene of *S. equi*. Thus, VicK is not essential in *S. equi*. However, the Δ*vicK* mutant is attenuated in virulence in both mouse models of subcutaneous and intranasal *S. equi* infections, indicating that VicRK is important to virulence. The results provide the further evidence for the importance of VicRK to virulence of Gram-positive pathogens.

*S. equi* Δ*vicK* mutant does not grow as well as the wild-type strain in both THY and blood, suggesting that the *vicK* deletion causes defect in growth, a plausible reason that likely contributes to the attenuation of *S. equi* virulence in the mouse infection models. This suggestion is further supported by the observations that both the wild-type and Δ*vicK* mutant strains are resistant to phagocytosis by PMNs, which suggest that VicRK is not required for the evasion of *S. equi* to the innate immunity. As introduced earlier, the various phenotypes have been described for the *vicRK* mutants of the various pathogens. Whether the phenotypes are specific to particular organisms is not known. The phenotypes of the *S. equi* Δ*vicK* mutant are similar to those of the *S. pyogenes* Δ*vicK* mutant [6], suggesting that the growth defect phenotype may be a common feature of the *vicRK* mutants of various Gram-positive pathogens.

The Δ*vicK* mutant appears to possess the properties of a potential live vaccine. First, it is attenuated in virulence in the mouse infection models. Secondly, Δ*vicK* inoculation protects mice against subsequent infection with wild-type *S. equi*. Thirdly, most of the mice with intranasal Δ*vicK* infection produce mucosal IgA and systemic IgG specific to protective antigen SeM. However, whether Δ*vicK* can be an effective live vaccine and whether the Δ*vicK* mutant has any advantages over the current live *S. equi* vaccine require the test of the mutant in horses since *S. equi* does not naturally infect mice. We hope to perform this expensive test in future when funds are available

## References

[1] Hoch JA (2000). Two-component and phosphorelay signal transduction. Curr Opin Microbiol.

[2] Fabret C, Hoch JA (1998). A two-component signal transduction system essential for growth of Bacillus subtilis Implications for anti-infective therapy. J Bacteriol.

[3] Martin PK, Li T, Sun D, Biek DP, Schmid MB (1999). Role in cell permeability of an essential two-component system in Staphylococcus aureus. J Bacteriol.

[4] Lange R, Wagner C, de Saizieu A (1999). Domain organization and molecular characterization of 13 two-component systems identified by genome sequencing of Streptococcus pneumoniae. Gene.

[5] Throup JP, Koretke KK, Bryant AP (2000). A genomic analysis of two-component signal transduction in Streptococcus pneumoniae. Mol Microbiol.

[6] Liu M, Hanks TS, Zhang J (2006). Defects in ex vivo and in vivo growth and sensitivity to osmotic stress of group A Streptococcus caused by interruption of response regulator gene vicR. Microbiology.

[7] Kadioglu A, Echenique J, Manco S, Trombe MC, Andrew PW (2003). The MicAB two-component signaling system is involved in virulence of Streptococcus pneumoniae. Infect Immun.

[8] Senadheera MD, Guggenheim B, Spatafora GA (2005). A VicRK signal transduction system in Streptococcus mutans affects gtfBCD gbpB and ftf expression biofilm formation and genetic competence development. J Bacteriol.

[9] Ng WL, Kazmierczak KM, Winkler ME (2004). Defective cell wall synthesis in Streptococcus pneumoniae R6 depleted for the essential PcsB putative murein hydrolase or the VicR (YycF) response regulator. Mol Microbiol.

[10] Echenique JR, Trombe MC (2001). Competence repression under oxygen limitation through the two-component MicAB signal-transducing system in Streptococcus pneumoniae and involvement of the PAS domain of MicB. J Bacteriol.

[11] Mohedano ML, Overweg K, de la Fuente A (2005). Evidence that the essential response regulator YycF in Streptococcus pneumoniae modulates expression of fatty acid biosynthesis genes and alters membrane composition. J Bacteriol.

[12] Timoney JF (1993). Strangles. Vet Clin North Am Equine Pract.

[13] Harrington DJ, Sutcliffe IC, Chanter N (2002). The molecular basis of Streptococcus equi infection and disease. Microbes Infect.

[14] Mukhtar MM, Timoney JF (1988). Chemotactic response of equine polymorphonucelar leucocytes to Streptococcus equi. Res Vet Sci.

[15] Timoney JF (2004). The pathogenic equine streptococci. Vet Res.

[16] Timoney JF, Artiushin SC, Boschwitz JS (1997). Comparison of the sequences and functions of Streptococcus equi M-like proteins SeM and SzPSe. Infect Immun.

[17] Anzai T, Timoney JF, Kuwamoto Y, Fujita Y, Wada R, Inoue T (1999). In vivo pathogenicity and resistance to phagocytosis of Streptococcus equi strains with different levels of capsule expression. Vet Microbiol.

[18] Woolcock JB (1975). Immunity to S equi. Aust Vet J.

[19] Hanks TS, Liu M, McClure MJ, Lei B (2005). ABC transporter FtsABCD of Streptococcus pyogenes mediates uptake of ferric ferrichrome. BMC Microbiol.

[20] White-Owen C, Alexander JW, Sramkoski RM, Babcock GF (1992). Rapid whole-blood microassay using flow cytometry for measuring neutrophil phagocytosis. J Clin Microbiol.

[21] Lei B, Liu M, Chesney GL, Musser JM (2004). Identification of new candidate vaccine antigens made by Streptococcus pyogenes Purification and characterization of 16 putative extracellular lipoproteins. J Infect Dis.

